# Towards an Urban Vibrancy Model: A Soundscape Approach

**DOI:** 10.3390/ijerph15081712

**Published:** 2018-08-10

**Authors:** Francesco Aletta, Jian Kang

**Affiliations:** UCL Institute for Environmental Design and Engineering, The Bartlett, University College London (UCL), Central House, 14 Upper Woburn Place, London WC1H 0NN, UK; f.aletta@ucl.ac.uk

**Keywords:** soundscape, environmental sounds, quietness, vibrancy, acoustic environments, urban sound planning

## Abstract

Soundscape research needs to develop predictive tools for environmental design. A number of descriptor-indicator(s) models have been proposed so far, particularly for the “tranquility” dimension to manage “quiet areas” in urban contexts. However, there is a current lack of models addressing environments offering actively engaging soundscapes, i.e., the “vibrancy” dimension. The main aim of this study was to establish a predictive model for a vibrancy descriptor based on physical parameters, which could be used by designers and practitioners. A group interview was carried out to formulate a hypothesis on what elements would be influential for vibrancy perception. Afterwards, data on vibrancy perception were collected for different locations in the UK and China through a laboratory experiment and their physical parameters were used as indicators to establish a predictive model. Such indicators included both aural and visual parameters. The model, based on Roughness, Presence of People, Fluctuation Strength, Loudness and Presence of Music as predictors, explained 76% of the variance in the mean individual vibrancy scores. A statistically significant correlation was found between vibrancy scores and eventfulness scores, but not between vibrancy scores and pleasantness scores. Overall results showed that vibrancy is contextual and depends both on the soundscape and on the visual scenery.

## 1. Introduction

The quality of the acoustic environments of modern cities is becoming a growing concern at a global scale. When such quality is poor because of (among other issues) high exposures to unwanted sounds, there will likely be noise pollution, which has been recognised as an element “affecting quality of life and well-being and (…) as an important public health issue” [1]. At different levels, noise issues are the object of attention of several groups with potentially competing interests towards the acoustic environment, including citizens, companies, policy-makers, local authorities, and planning and design professionals. The policy framework for this topic in the Member States of the European Union is provided by the so-called “Environmental Noise Directive (END)” [2], which brings guidance on the “assessment and management of environmental noise”. It is now generally acknowledged that the management of the urban acoustic environments can no longer rely on a mere noise control or acoustic retrofitting approach [3,4,5,6] and it should extend to a broader concept of “urban sound planning” [7]. A number of local authorities around Europe embraced this cause and tried to implement several actions into their policies, aimed at enhancing the environmental sound quality in a “proactive”, rather than a “reactive”, way (e.g., [8,9,10]).

This shift towards a quality paradigm calls for further attention on how acoustic environments are perceived. Within this framework, the soundscape philosophy plays a key role. Soundscape is the perceptual construct deriving from the human experience and understanding of any acoustic environment, in context [11]. Ever since its appearance as a research field, soundscape soon became a relevant topic for planners and designers questioning the “sonic identity” of cities and how this would match their “visible” reality [12,13].

In recent years, soundscape research has been going through rapid expansion, with international experts and research groups aiming at standardizing definitions, methods and analysis procedures [11,14,15,16]. This is possibly due to the scientific community′s will to provide policy-makers and practitioners with operative tools (i.e., predictive models). There is a current lack of soundscape descriptors and indicators, which has been previously identified as a gap to fill, in order to introduce the soundscape approach into the urban realm′s management (e.g., [17,18,19]).

Aletta et al. [20] recently reviewed the main soundscape descriptors and indicators, where “descriptors” are meant as “measures of how people perceive the acoustic environment” and “indicators” are “measures used to predict the value of a soundscape descriptor”. The review showed that overall descriptors referred either to single dimensions of soundscape appreciation (e.g., calmness), or to soundscape holistically (e.g., soundscape quality). It also pointed out that many descriptors have a focus on calmness or similar constructs (e.g., tranquillity, quietness). A possible explanation for this is that the Environmental Noise Directive explicitly urged the Member States to identify and preserve quiet areas but provided little guidance on the criteria to consider. Thus, a lot of research efforts went in that direction (e.g., [21,22,23,24,25,26,27]). Consequently, the European Environmental Agency (EEA) released a good practice guide on quiet areas, where the soundscape methodology is officially endorsed for the first time at an international policy level [6]. The EEA review also includes the tranquillity rating tool, developed by Watts and his colleagues, which considers the ratio of greenery features in a scene and sound pressure level as main predictors (i.e., indicators) for the tranquillity descriptor.

Nonetheless, it is worth noticing that a soundscape descriptor related to a single dimension should be relevant for the investigated context. Would it make sense to use a tranquillity descriptor to assess the soundscape quality of Piccadilly Circus in London? Possibly not. But this doesn’t necessarily mean that such a place is not able to elicit positive soundscapes. Local authorities and planners may need to work out the soundscape quality of places where “the quieter, the better” strategy might not be the best option [28,29].

**Figure 1:**
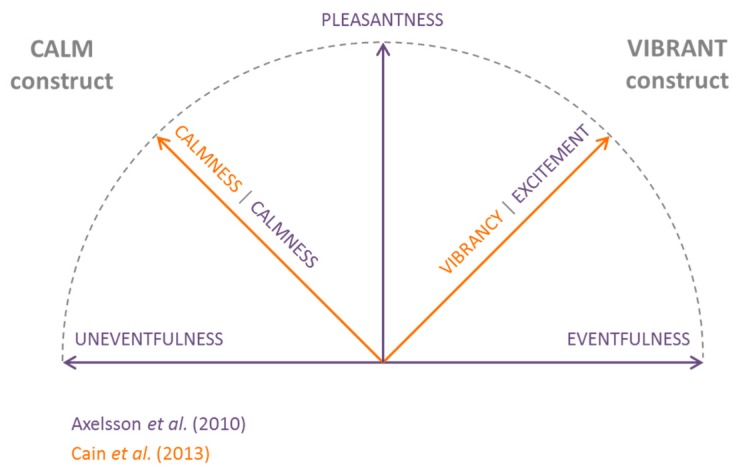
Schematic representation of the models for soundscape characterisation. Figure adapted from Axelsson et al. [30] and Cain et al. [31].

Models for soundscape characterisation have been proposed by Axelsson et al. [30] and Cain et al. [31]. These seem to converge towards two-dimensional models of perceived affective quality and provide for the most comprehensive information about soundscape appreciation [20]. Axelsson et al.’s model is defined by two orthogonal factors “Pleasantness” and “Eventfulness”, which are located at a 45° degrees rotation from the second set of orthogonal factors “Calmness” and “Excitement”. According to this model, a soundscape that is both pleasant and eventful will be “exciting”, whilst a soundscape that is both pleasant and uneventful will be “calm”. Likewise, the model by Cain et al. includes two orthogonal factors, i.e., “Calmness” and “Vibrancy” (instead of “Excitement”). Figure 1 summarises the two models and shows how they seem to agree on the fact that, for a soundscape to be positive (i.e., pleasant), this should either be calm or vibrant, and these two factors are not straightforwardly related to sound levels [31]. The first factor relates to the possibility of achieving quiet and restorative soundscapes (i.e., the calm construct); the latter is more oriented to the potential of offering actively engaging soundscapes (i.e., the vibrant construct). Both models point out one aspect of soundscape, namely the “vibrancy” or “excitement”, which has not been previously covered by descriptor-indicator(s) models [20]. Within the framework of this research we will refer to the term “vibrancy”. This is ultimately the descriptor that is being sought in order to characterise (and eventually plan and design) in a more relevant way the soundscape quality of pleasant and eventful places (like Piccadilly Circus, for instance). Thus, this work acknowledges the need to develop a predictive model for vibrancy based on a set of corresponding physical indicators, as a tool to be used by planners and designers, in contexts where such a descriptor is likely to be relevant.

It is worth pointing out at this stage that the soundscape (or rather, the acoustic environment) of a place should not be treated in isolation or designed independently of other factors of an urban environment. From a planning and design point of view, measuring the vibrancy of an acoustic environment as an independent factor would be a pointless exercise, since in the real world it does not seem likely that a place (as a whole) would be vibrant while its soundscape (alone) would be not. The research on tranquillity of Watts and colleagues [22,23,24,25] faced a similar issue, where they did not assess the tranquillity of a place as a separate dimension from its soundscape, but rather investigated the tranquillity of the place as a whole, including both visual and acoustic aspects. Such methodological approach is typical in soundscape studies, as soundscape research aims at considering several environmental components and their interactions in contest in a holistic way, rather than treating them as unrelated parts of the built environment.

The main aims of this study are: (1) to investigate how the vibrancy construct is overall understood and how it could be relevant for soundscape research; (2) to establish a predictive model (i.e., identifying indicators) for a “vibrancy” descriptor; and (3) to further explore the relationships between the vibrant, eventful and pleasant constructs in soundscape. In order to address the first aim, a group interview was carried out with acousticians and designers to explore the vibrancy construct so to formulate a hypothesis on what elements would be influential for vibrancy perception. For the second aim, a laboratory experiment was carried out to collect soundscape data on vibrancy perception and to establish a predictive model using indicators derived from the group interview stage. While both the descriptors and indicators considered in this study might be already known in soundscape literature, it was considered useful to perform the group interview and use data derived from it to inform the second part of the study (laboratory experiment). To some extent, this helps to limit a potential “experimenter’s bias”, which could have occurred if the indicators were selected on a totally arbitrary basis.

## 2. Materials and Methods

**Figure 2:**
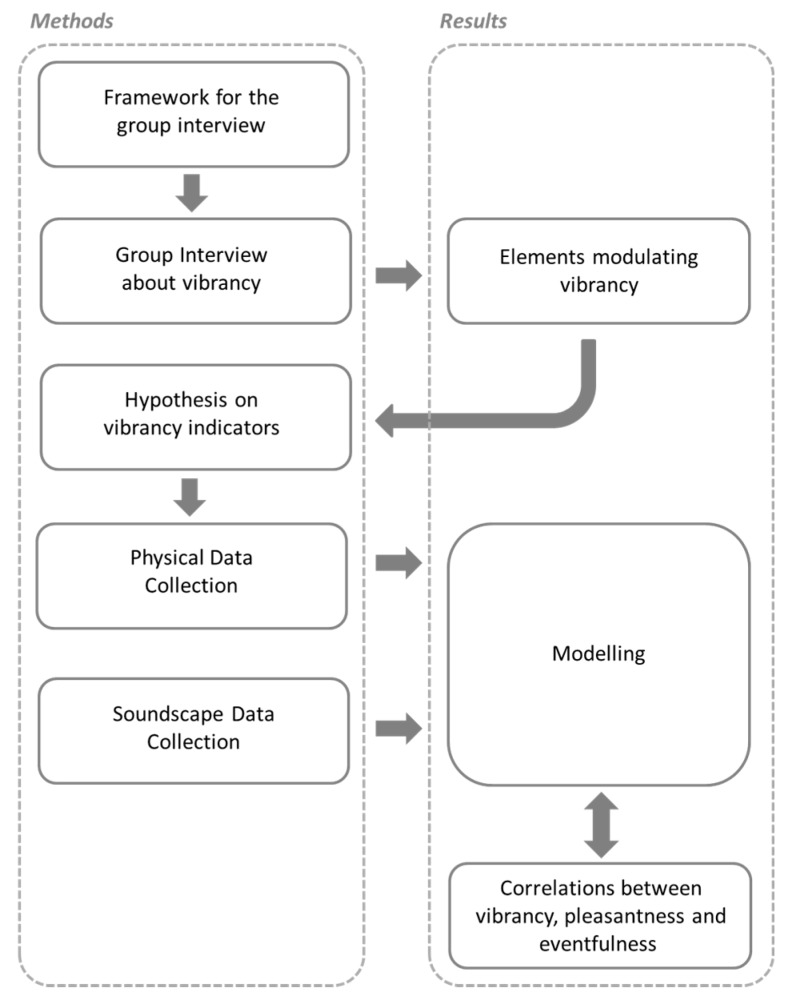
Schematic representation of the methodological workflow considered within the current research.

Despite sounding like a relatively familiar concept, little is known about how “vibrancy” is defined or understood, under a planning and design perspective. Hence, the first stage of the work aimed to establish a framework that would inform the group interview stage, which was in turn supposed to provide information about what elements people perceive to be relevant for the vibrant construct. Having extrapolated the likely vibrancy influential elements from the group interview, it was possible to make a hypothesis on potential physical metrics (i.e., indicators) that could be proxy for the abovementioned elements. For the experimental stage, these metrics would then be computed for the collected recordings and used as predictors of the vibrancy descriptor in a statistical model. Figure 2 shows the methodological approach adopted in the current research. The flowchart reflects that, since a number of assumptions needed to be made, the workflow did not follow a linear development. Boxes correspond to sub-sections of the paper, as addressed in the main sections, namely Methods and Results.

This study was granted ethical approval by the Research Ethics Committee of the School of Architecture of the University of Sheffield, UK (former institution of the authors; this is where the study originally started), with approval letter ref. 007015 (01.12.2015). All participants, both for the group interview and the audio-visual experiment presented below, provided informed consent.

### 2.1. Framework for the Group Interview

As a preparatory work for the group interview stage, the vibrancy concept was explored in urban studies and soundscape literature so to prepare a framework to inform questions and aspects to ask people about, when it comes to their perception and understanding of vibrancy.

The attribute “vibrant” is usually referred to something that is “full of energy and life” [32]. In urban studies, it is not a new concept and is conventionally associated with downtowns and cities (e.g., [33,34]), and environments that “facilitate non-motorized transportation, connect activities in space, promote health and equity, emphasizes diverse land uses, preserve environmental resources, and encourage social exchange in the public realm.” (ref. [35], as cited in [36]). Braun and Malizia [36] developed a composite vibrancy index to describe the vibrancy of 48 downtown areas, taking into account urban compactness, density, regional and local connectivity, destination accessibility, land use, and social diversity. They found that vibrancy is associated with more favourable population-level health and safety outcomes in central urban environments. Such findings might be particularly relevant under an urban design perspective, and support the cities’ efforts at a policy level to generate “more vibrant centres in support of innovation and economic development”.

In soundscape studies, the vibrancy concept has been addressed in several studies (e.g., [30,31,37]) and there it has been suggested that the vibrant construct is positively associated to the pleasantness dimension of soundscapes (e.g., [38]). Davies et al. [37] concluded that soundscape vibrancy is related to two auditory aspects: organisation of sounds and changes over time. These two aspects can be in turn described by two qualitative dimensions, namely: cacophony-hubbub and constant-temporal, which can elicit a vibrancy response in the listener.

However, Hall et al. [38] showed that the association between vibrancy responses and conventional psycho-acoustic metrics (e.g., loudness, roughness, fluctuation strength and sharpness, or metrics based on averaged spectral shape) is not straightforward. In their experimental study, even though some psycho-acoustic metrics significantly correlated with vibrancy responses, the final model only explained 3% of the overall variance in the data. This suggests that when it comes to perceived soundscape vibrancy, people might be affected by other non-acoustic factors. Thus, it seems fair to investigate further what indicators are likely to be relevant for such a descriptor.

### 2.2. Group Inteview about the Vibrancy Concept

Given the sociological nature of soundscape research, semi-structured interview techniques, like group interviews and focus groups, are often considered as a suitable method for collecting data about the perception of sound environments or some of their components (e.g., [39,40]). Within the framework of this research, there was a need to investigate how the concept of vibrancy of a place is overall understood, so to consider what factors could be relevant to provide a “vibrant urban environment” to people. For this purpose, a group interview was organised. Seven postgraduate students, doctoral students and researchers in architecture, acoustics and planning were invited to take part. The rationale for participants’ selection was having a group with a relatively common background, but not necessarily the same attitude towards a topic [41], as well as participants who were likely to provide useful insights into the vibrancy perceptual attribute, under a planning and design perspective.

The session took place in a meeting room of the School of Architecture of the University of Sheffield. Two experimenters coordinated the discussion asking open questions, and participants had the opportunity to express their views, exchange ideas and agree on a number of points. The session lasted approximately 45 min and it was audio-recorded for further semantic analysis (consent had been previously collected from participants for this purpose). The questions were: “What does vibrancy mean for you?”, “Overall, is vibrancy something good for you?”; “What would the opposite of vibrant be?”; “What elements contribute to make a vibrant urban environment for you?”; “Can you give me an example of an urban environment that is/is not very vibrant?”; “How would a vibrant urban environment sound like?”; “How would a vibrant urban environment look like?”. It is important to highlight that the concept of vibrancy, in general, might be understood differently across different cultures or simply personal backgrounds. While the aim of this study was establishing a preliminary vibrancy model, more studies targeting specific cultures and countries might be useful.

### 2.3. Hypothesis on Vibrancy Indicators

The results of the group interview stage will be discussed in Section 3.1, but for the sake of clarity they are briefly anticipated here, as they serve as basis for the hypothesis on the vibrancy indicators. Overall, people agreed that the elements modulating the vibrancy perception are related both to the aural (i.e., loudness, variability, human voices, and music) and the visual (i.e., people and activity) domain.

**Figure 3:**
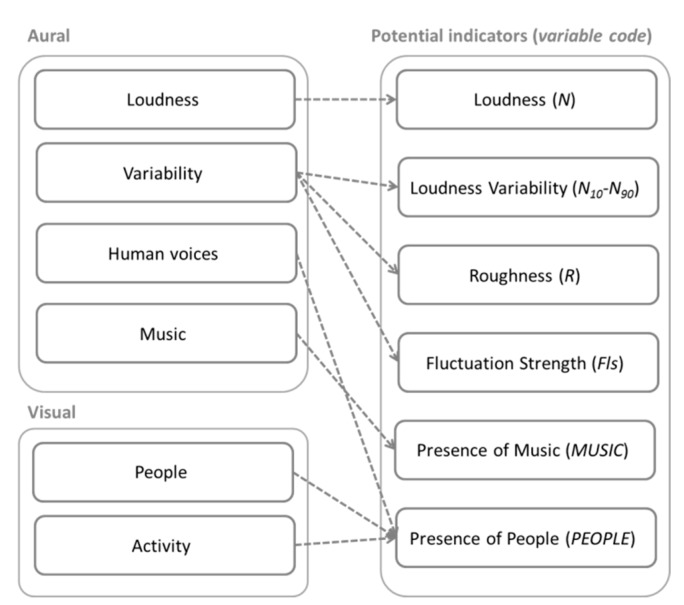
Schematisation of variables hypothesised for the elements of the group interview.

In order to establish a vibrancy model, a hypothesis was made about what physical indicators (i.e., measurable quantities) could potentially be effective predictors of the perceptual elements derived from the group interview [20]. This resulted in the following parameters: Loudness (*N*), Loudness Variability (*N*_10_*–N*_90_), Roughness (*R*), Fluctuation Strength (*Fls*), Presence of Music (*MUSIC*) and Presence of People (*PEOPLE*). The rationale for the parameters’ selection was finding the best physical proxy for the perceptual descriptors. Since soundscape is a complex and multi-layered construct, it was assumed that different indicators might refer to the same perceptual elements; likewise, a single perceptual element might well be represented by different indicators, as schematised in Figure 3. The metrics are briefly described below.

The loudness of a sound reflects the intensity sensation of the energy content of sound on the human hearing. In perceptual studies it is usually preferred to other metrics like sound pressure level, as it is considered to better represent how the human ear perceives sounds [42]. There are several methods for calculating the loudness. This study will refer to Loudness (*N*) as defined in Fastl and Zwicker [43] and its values are expressed in *sones*. In order to account for Loudness changes over time, statistical levels of Loudness (i.e., levels exceeded for an *N*_x_ percentage of time, with respect to the reference period) will be considered. Thus, the Loudness Variability over time (*N*_10_–*N*_90_) can be represented by the difference between the Loudness peak values (*N*_10_) and the Loudness background values (*N*_90_).

Roughness (*R*) is a metric related to the perceptual effect of fast amplitude modulation of a sound (15–300 Hz) and it is measured in *aspers* [43]. Likewise, Fluctuation Strength (*Fls*) is a metric related to slower (up to 20 Hz) amplitude modulation of a sound and it is measured in *vacils* [43]. Both these metrics are usually considered to be representative of a sound’s temporal variation [44].

For the purposes of this study, Presence of People (*PEOPLE*) was defined as a numerical variable and computed for a site by summing the persons represented in a scene; thus it is expressed in integers. On the other hand, Presence of Music (*MUSIC*) was defined as a binary variable, considering whether music can (1) or cannot (0) be heard at any moment during a reference auditory stimulus.

### 2.4. Physical Data Collection

Audio-visual data were collected from 46 locations across England and China using a Canon EOS 500D camera to record the visual information and a binaural headset (in-ear 1/8′′ DPA microphones) connected to an Edirol R44 portable recorder to capture the auditory and acoustic data [45]. The locations chosen for the study were selected from the city centre of Sheffield and Doncaster (UK), and Beijing and Tangshan (China). The reasons for this were: (1) to provide a wide range of urban environments with different activities (e.g., commercial, residential, service areas); (2) to sample stimuli from the entire two-dimensional soundscape model so that, for instance, also calm or chaotic or monotonous environments are considered (and not only vibrant ones); (3) to provide different cultural and social backgrounds between European and Asian contexts; and (4) to consider cities that could be representative of different urban sizes (compared to the corresponding countries). Table 1 reports the selected locations for data collection and the corresponding main urban activities as noted during the on-site campaign.

**Figure 4:**
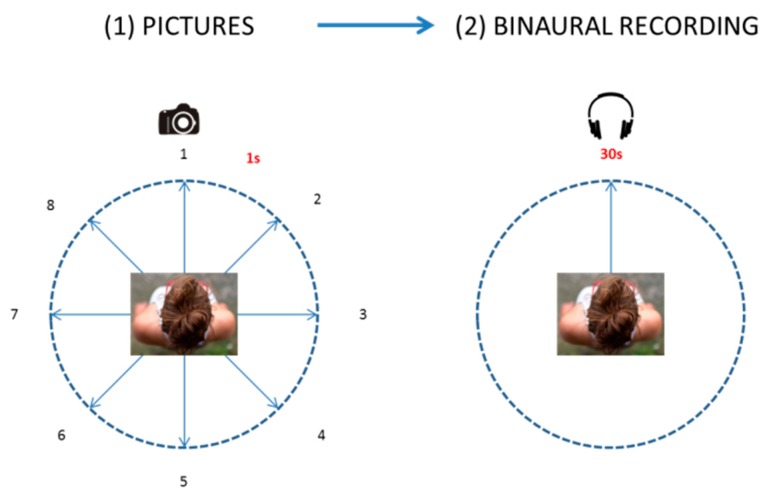
Scheme representing the procedure for audio-visual data collection.

At each location, for visual data, an operator swept clockwise taking a picture on a normal setting every 45° (with approximately one-second intervals) so to have eight contiguous pictures covering a 360° view in the horizontal plane, at a height of 1.70 m [25]. Immediately after that, the operator performed a 30-s audio-recording with the binaural headset, with a steady head orientation. The audio-visual recording procedure is summarised in Figure 4.

With the purpose of providing input data for the modelling stage, the indicators described in Section 2.3 were calculated for each of the 46 sample locations. Table 2 reports the values of the different variables for each of the 46 locations considered in the study. The psychoacoustic indicators were computed using the software Artemis v.11 [46], while the other variables where computed manually through audio-visual inspections, and cross-validated by two research students.

### 2.5. Soundscape Data Collection

According to the conceptual framework for the development of soundscape predictive models proposed in Aletta et al. [20], after the physical characterisation of the acoustic (or visual) environment, it is necessary to gather individual data about perception. For this purpose, a laboratory experiment was carried out to collect responses on the perceived vibrancy of the investigated urban environments. Axelsson et al. [30] define vibrant (or exciting) the soundscape that is both pleasant and eventful. Thus, individual responses were collected also for the latter attributes, in order to further validate the perceptual information.

Thirty-five undergraduates and postgraduates and staff members at the University of Sheffield, 18 to 46 years old, took part in the experiment (19 women, 16 men; M_age_ = 26.5 years, SD = 5.8). Participants were selected from a group of 200+ persons who completed an online survey circulated via the established email list for research volunteers at the University of Sheffield. The online survey was designed to achieve a varied sample of participants in terms of gender, age and ethnic origin. The 35 participants who completed the experiment received 5 GBP as a token of appreciation for volunteering in the experiment.

Forty-six videos (30 s) were used for this experiment, corresponding to the 46 locations where physical data were collected. The auditory part of the video consisted of the 30-s binaural recordings, as collected on site. The visual part consisted of a transition of the eight pictures, (from picture 1 to picture 8, as shown in Figure 4), for 3.75 s each [22]. The equipment used for the experiment consisted of a 16″ laptop (HP EliteBook 850, Hewlett-Packard, Palo Alto, CA, USA), and a pair of open, circum-aural headphones (HD 558, Sennheiser, Wedemark, Germany). The audio part of the video was played back at the original sound-pressure level as recorded on site (Type 4231 calibrator, Brüel & Kjær, Nærum, Denmark).

The experiments were carried out in a silent meeting room (background noise <25 dBA) at the School of Architecture of the University of Sheffield. Participants took part individually. Upon arriving, they were asked to sign the informed consent and report if they had a normal or corrected to normal hearing and vision. Some demographic information was collected for descriptive purposes. Sitting at a desk with the laptop, participants were given the headphones and the experiment started. The stimuli were presented via an online platform in a randomised sequence for each participant, so to limit potential order effects. Participants were only allowed to listen to the recordings once. The experimental sessions lasted between 30 and 40 min.

After each scenario, participants were asked to answer three questions on a ten-point scale ranging from “not at all” (0) to “extremely” (10): (a) “Overall, how vibrant was the sound environment that you have just experienced?”; (b) “Overall, how eventful was the sound environment that you have just experienced?”; (c) “Overall, how pleasant was the sound environment that you have just experienced?”. Since “eventful” and “vibrant” are attributes that are likely to generate ambiguity, participants were previously instructed to consider eventful a sound environment that “is related to the presence of significant events that characterize the sound environment, defining it as a non-flat context”, and to consider vibrant a sound environment relating to “excitement, creating a soundscape that is ‘full of life’ and activating”. While participants were tutored to consider the vibrancy of the place “holistically” (i.e., both aurally and visually), the questions explicitly mentioned the “sound environment” so that the sample would pay particular attention to the soundscape construct, which in such complex audio-visual stimuli could be possibly disregarded in favour of vision.

Since the meaning attributed to “vibrant” was crucial for the experiment, particular attention was given to this concept to avoid that it could be confused with the abovementioned “eventful”. When the meaning was not clear, participants were offered synonyms for vibrant, such as “exciting” or “lively”. This is a common practice in behavioural science, where multiple attributes are typically used to define an index for the underlying construct, since this increases the quality of the data, and the likelihood of valid results [47].

## 3. Results

Results are divided in three sub-sections. Section 3.1 reports the output of the group interview about vibrancy, which has been already referred in Section 2.3 to state the hypothesis about the potential vibrancy indicators. Section 3.2 establishes the predictive model for the vibrancy descriptor, based on the perceptual and physical data. Section 3.3 eventually explores further associations between vibrancy and its underpinning dimensions (i.e., eventfulness and pleasantness).

### 3.1. Elements Modulating Vibrancy

The transcription of the group interview was coded using general concepts that could help to define how vibrancy is understood in the urban realm [48]. This thematic analysis refers to the “grounded theory”, which is becoming an increasingly important methodological approach in soundscape studies [40,49]. According to this method, the investigation should start with a (set of) question(s) and collection of qualitative data (the transcription of the group interview, in this case). Recurring concepts are then tagged with “codes” in an iterative process; codes are then grouped into concepts, and then into categories. The final categories are those likely to become the basis for a new framework/theory.

Overall, the group agreed that vibrancy is related to a pleasantness dimension (e.g., “To me, [vibrancy] implies positive feelings, so if an area is vibrant it implies that it makes you feel good and gets yourself in a state of excitement”), which is consistent with previous literature [30,48], and it might be affected by people’s preconceptions or background about a specific urban context (e.g., “I think your preconceptions as well can influence. If you have heard an area is exciting, I think you bring your own biases and preconceptions about the area as well and get yourself in that mood” or “…you might hear from some friend that this area is very cool, a lot of bars etc., you should go…and this might influence your perception of vibrancy”).

Regarding the elements that contribute to a vibrant perception of an urban context, the thematic analysis of the group interview transcription revealed that there are a number of core elements (codes), which can be in turn sorted into two main categories, namely: aural factors and visual factors. Table 3 reports the main factors that emerged from the group interview, that participants considered being relevant for the vibrancy of an urban environment.

### 3.2. Modeling Vibrancy

A stepwise linear regression analysis was conducted, using the vibrancy scores (individual values averaged across the 35 participants, for each site) as dependent variables and the set of six parameters as independent variables (SPSS 22 for Windows, IBM Corporation, Armonk, NY, USA). The model explained 75.9% of the variance in the dependent variable. The strongest predictors of vibrancy were *R* (*t* = 6.314, *p* < 0.001), *PEOPLE* (*t* = 4.447, *p* < 0.001), *Fls* (*t* = 4.163, *p* < 0.001), *N* (*t* = −4.358, *p* < 0.001), and *MUSIC* (*t* = 3.123, *p* = 0.003); (*F*_5, 40_ = 25.21, *p* < 0.001, R^2^ = 0.76). The sixth variable (*N*_10_–*N*_90_) was excluded by the regression algorithms: this is further discussed in Section 4.1.

**Figure 5:**
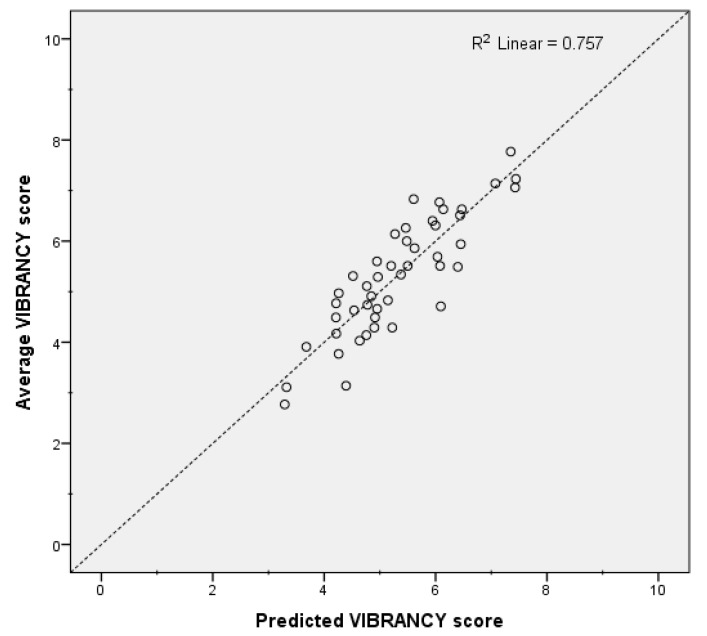
Predicted vibrancy scores vs. actual vibrancy scores (average across all participants).

Table 4 shows that *R* explained 39.4% of the variance in vibrancy. When controlling for this variable, *PEOPLE* explained an additional 14.6% of the variance. Likewise, *Fls*, *N*, and *MUSIC* explained an additional 6.7%, 9.3% and 5.9% of the variance, accordingly. Overall, the positive relationship between vibrancy and *R* shows that there was more rapid amplitude modulation associated with the acoustic environments interpreted as vibrant. For the visual aspects, the more people in the scene, the more vibrant the environment was perceived. Figure 5 shows the strength of the relationship between the average vibrancy scores collected during the listening experiment, and those predicted by the vibrancy model proposed above.

Strong collinearity between variables was dismissed after checking for the variance inflation factor (VIF) of the predictors used for the vibrancy model (VIF values: *R*, 1.936; *PEOPLE*, 1.594; *Fls*, 1.402; *N*, 2.934; *MUSIC*, 1.260).

As a further check on the reliability of the model, a filter variable was created in the original database to randomly select a subset of approximately 75% of the sample. This subset and the other covering the remaining 25% of the dataset were used to calibrate the model. The linear regression algorithm was run again using 75% of the dataset and achieved an explained variance of approximately 73%, compared to the 76% of the full dataset. Afterwards, a bivariate correlation analysis between the vibrancy scores and the predicted vibrancy values of the models from the two subsets was performed. The Pearson’s product-moment correlation coefficients for the two subsets were similar: *r*(34) = 0.847, *p* < 0.001 for the subset of the 75% of the sample and *r*(12) = 0.889, *p* < 0.001 for the subset of the remaining 25% of the sample. Thus, severe issues of overfitting were deemed to be negligible.

### 3.3. Correlation between Vibrancy, Pleasantness and Eventfulness

**Figure 6:**
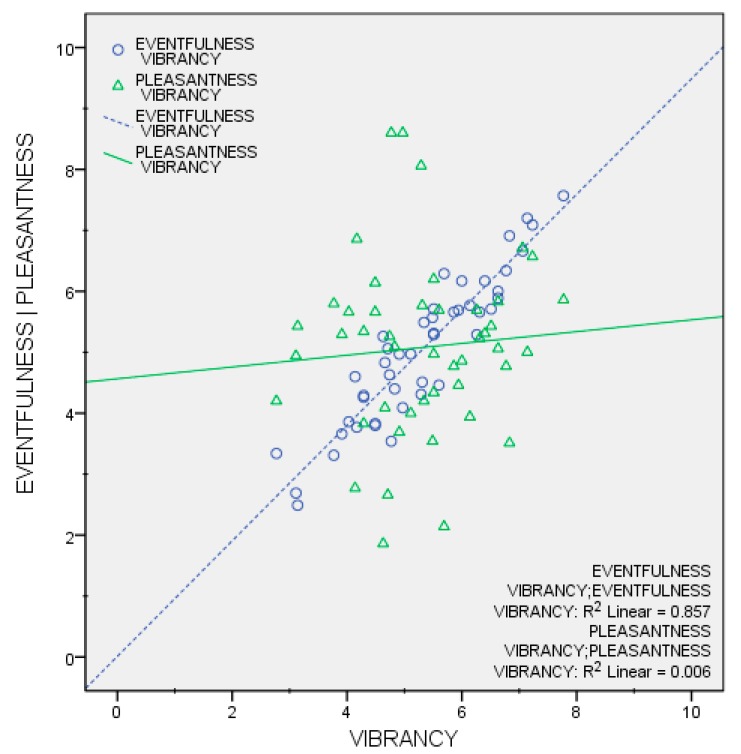
Scatterplots for mean vibrancy scores vs. mean eventfulness scores and mean vibrancy scores vs. mean pleasantness scores.

In order to provide further insights into vibrancy perception, two Pearson product-moment correlation coefficients were computed to assess the relationships between the mean vibrancy scores and the mean pleasantness scores, and the mean vibrancy scores and the mean eventfulness scores. There was a strong positive correlation between vibrancy and eventfulness, *r*(46) = 0.926, *p* < 0.001. However, no statistically significant correlation was observed between vibrancy and pleasantness: *r*(46) = 0.079, *p* = 0.604. Figure 6 summarises these results.

Such lack of correlation was further explored, while controlling for the “main urban activity” (as per in Table 1) variable. No statistically significant correlation between vibrancy and pleasantness emerged in this case either, for most of the urban activity categories: tertiary, *r*(6) = −0.417, *p* = 0.411; entertainment, *r*(7) = 0.614, *p* = 0.143; commercial, *r*(21) = 0.405, *p* = .069; residential, *r*(8) = −0.228, *p* = 0.588. The only exception was the strong and statistically significant negative correlation between vibrancy and pleasantness for the urban activity category green areas: *r*(4) = −0.985, *p* = 0.015. This was somewhat expected since green areas, when eliciting pleasantness, are most likely assessed as calm (and not vibrant) [22,23,24,25].

**Figure 7:**
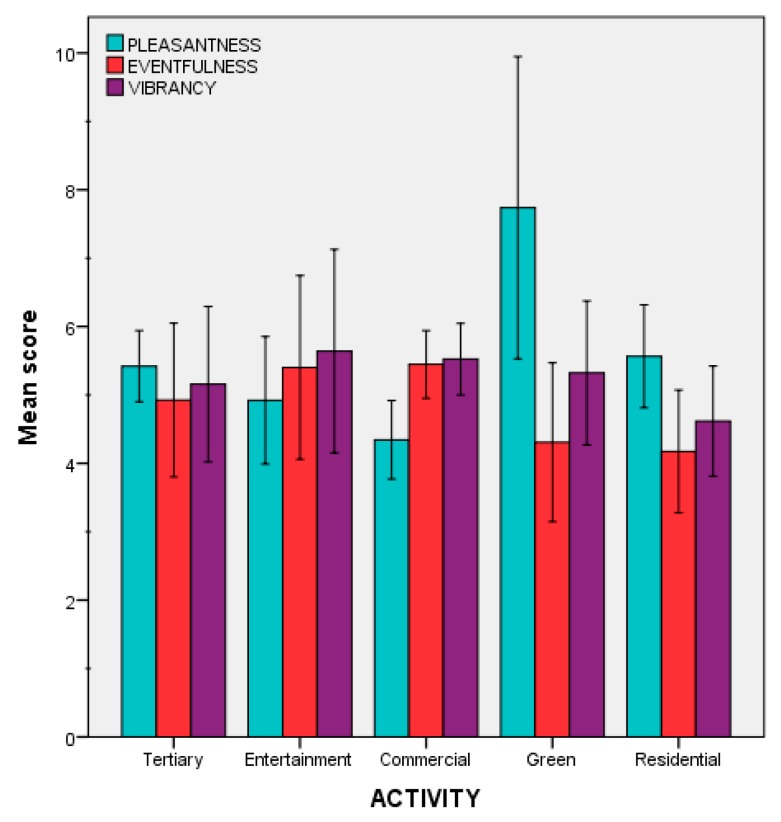
Mean scores for pleasantness, eventfulness and vibrancy (Error bars: 95% CI).

This suggests that while the association between vibrancy and eventfulness coming from previous studies [30] is perceptually appreciated in this experiment, pleasantness might be more affected by the contextual information (e.g., visual factors). To support this hypothesis, a one-way between subjects ANOVA was conducted to compare the effect of the main urban activity (as reported in Table 1) taking place in each of the 46 locations of this study (as a proxy for context) on the mean pleasantness scores. There was a general significant effect of the context on pleasantness scores, *F*(4, 41) = 8.597, *p* < 0.001. A post hoc Bonferroni test indeed revealed that, for the pleasantness scores, “green” locations (e.g., urban parks) significantly differed from all other contexts: “tertiary” (*p* = 0.024); “entertainment” (*p* = 0.002); “commercial” (*p* < 0.001); and “residential” (*p* = 0.027). Figure 7 reports the mean scores for the three variables considered in the laboratory experiments, where such differences can be observed.

Furthermore, a set of independent-sample *t*-tests was run to determine if there were differences in pleasantness, eventfulness or vibrancy between UK (Sheffield and Doncaster) and Chinese (Tangshan and Beijing) sites. No statistically significant differences emerged, for any of the three variables; pleasantness: UK (*M* = 5.50, *SD* = 1.19), China (*M* = 4.84, *SD* = 1.04), *p* = 0.059; eventfulness: UK (*M* = 5.49, *SD* = 1.17), China (*M* = 4.82, *SD* = 1.04), *p* = 0.052; vibrancy: UK (*M* = 5.56, *SD* = 1.19), China (*M* = 4.99, *SD* = 1.07), *p* = 0.102. This suggests that, at least for this experiment, the sample was not particularly influenced by the “cultural” content of the stimuli, either aurally (e.g., language of the voices heard, type of music, etc.) or visually (e.g., language of the shops’ windows, ethnicity of the people in the scene, etc.).

## 4. Discussion

The construct of vibrancy has been showed to be multi-dimensional and to rely on different sensory elements. While the physical characteristics and information content of the acoustic environment are certainly important, the group interview conducted in this study pointed out that other visual aspects might contribute to modulate vibrancy perception, which is in line with the holistic approach underpinning the soundscape theory [11]. Particularly, the presence of people, as both aural (i.e., human voices) and visual (i.e., groups or individuals within sight) sources, was regarded as a key component of the vibrancy experience. The presence of people is indeed likely to provide a social dimension that seems to be at the core of vibrancy perception, and previous studies reported that even the aural presence of humans alone can enhance the perceived safety of a place [50].

### 4.1. The Vibrancy Model

In a previous study based on a listening laboratory experiment, Hall et al. [38] proposed a predictive model for soundscape vibrancy, but they found that even though some acoustical and psycho-acoustical factors were significantly correlated with vibrancy scores, it was not possible to explain more than 3% of the model variance. The authors attributed this issue to individual differences in the listeners’ approach to soundscape rating or other non-acoustic factors. The point raised in this study is that also visual elements are crucial in vibrancy appreciation and when the auditory stimuli are presented together with the visual context, the listeners integrate the information coming from the aural and visual domain and report assessments that are better predicted by the physical indicators.

Roughness and Fluctuation Strength together accounted for more than 45% of the variance in vibrancy scores. To some extent this was expected, considering that these parameters are often related to the “impression” of a sound’s temporal variation [44], which is one of the elements emerged from the group interview. Interestingly, Roughness has usually been considered as a negative feature for “soundscape quality”, i.e., the rougher the acoustic environment, the poorer the soundscape quality [51]. Thus, this finding suggests that the same indicator might perform differently at predicting a single soundscape dimension, like vibrancy, rather than soundscape “holistically” (i.e., whether a soundscape is “good” or “bad”) [20,39,42].

**Figure 8:**
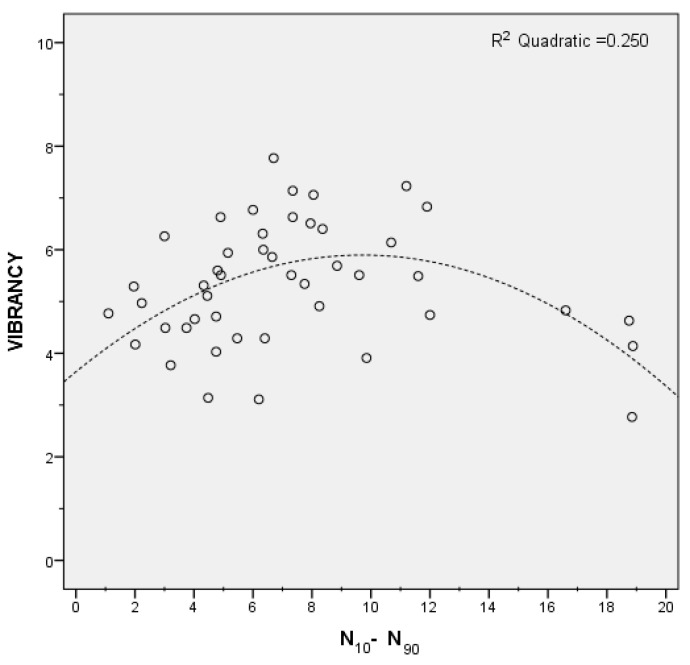
Scatter plot of the loudness variability (*N*_10_–*N*_90_) values vs. the mean vibrancy scores.

The loudness variability (*N*_10_–*N*_90_) indicator was excluded from the model by the stepwise linear regression algorithm. When plotting the mean vibrancy scores versus the *N*_10_–*N*_90_ values for the 46 investigated locations, it appears clearly that such a relationship is not linear, as reported in Figure 8. However, a quadratic fit for the loudness variability was found to explain 25% of the variance in vibrancy. Particularly, low and high loudness variability levels corresponded to low vibrancy, while moderate loudness variability increased vibrancy. A possible explanation for this is that, for a soundscape to be vibrant, loudness changes in time are relevant, but if these become overwhelming (e.g., like for acoustic environments dominated by traffic noise), the vibrant construct evolves into something different (possibly, chaotic, according to Axelsson et al. [30]).

Regarding the *PEOPLE* and *MUSIC* factors, it could be argued that they are oversimplified representations for a complex urban environment. However, there was a deliberate attempt for keeping these variables simple, so that the predictive model could potentially be implemented in future automatic monitoring systems, with limited computational resources.

### 4.2. Vibrancy, Pleasantness and Eventfulness

According to soundscape literature, vibrancy should be correlated with both eventfulness and pleasantness, and the latter two variables should be independent. The measurements of vibrancy gathered in this study correlated with eventfulness and not with pleasantness, seemingly suggesting that an eventfulness measurement was collected. However, as mentioned in Section 2.5, the participants of the audio-visual experiment were clear about the meaning of vibrant and eventful. The rationale for seeking correlations between vibrancy and eventfulness, and vibrancy and pleasantness, was indeed testing the theory developed by Axelsson et al. [30], stating that an “exciting” (or else, vibrant) soundscape is both eventful and pleasant. This was also confirmed by the information gathered during the group interview stage of this study. On the other hand, Hall et al. [38] in their study on psychoacoustic properties of urban soundscapes found no evidence for a relationship between the vibrant and pleasant constructs and concluded that these attributes are referred to independent dimensions. Nevertheless, the abovementioned studies [30,38] relied on audio-only laboratory experiments, and the group interview of this study addressed (soundscape) vibrancy perception “in theory”, while the participants of the audio-visual experiment looked at (vibrant) environments as a whole. That is, the visual information could not be disregarded. The results of the present study are somewhat in line with the findings of Hall et al. [38], as no correlation was found between vibrancy and pleasantness, but this should be considered in the broader understanding that the vibrancy construct is maybe too complex to be captured by auditory factors alone, and it could be highly affected by the contextual (e.g., visual) situation [52]. In order to confirm this outcome, it could be useful to perform further experiments including control conditions (e.g., audio-only or video-only stimuli) to gain a better understanding of the corresponding weights of the auditory and visual domains in the vibrancy construct. However, this was out of the scope of the present work, the primary aim of which was testing a predictive model.

It could still be meaningful to assess soundscape vibrancy in isolation from the context as some have done in the past, for mapping and assessment purposes, although this is less relevant if the purpose is to plan and design a (vibrant) place. Then, the soundscape cannot be treated separately, but must be approached as an integrated part of the place as a whole.

## 5. Conclusions

This paper aimed to provide further insights into the perceptual construct of vibrancy in soundscape studies and to provide a predictive model for the vibrancy descriptor using physical indicators. For this purpose a two-stage data collection was organised through a group interview and a laboratory experiment. Overall, the main conclusions of this study are:

- Vibrancy perception depends on both aural and visual cues, and the presence of people is relevant for both sensory domains.
- A vibrancy model based on Roughness, Presence of People, Fluctuation Strength, Loudness and Presence of Music as predictors, can explain up to 76% of the variance in the mean individual vibrancy scores.
- Within this audio-visual laboratory experiment, mean vibrancy scores resulted strongly correlated with mean eventfulness scores, but not correlated with mean pleasantness scores.

From a holistic perspective, this study suggests that the pleasantness dimension is contextual and highly dependent on the visual scenery. Taken together, the findings of this study show that there is room for the implementation of predictive models for new soundscape descriptors and these can be useful operative design tools within a broader urban sound planning framework.

## Figures and Tables

**Table 1:** Locations selected for data collection and main urban activity taking place there.

ID	City	Reference	Coordinates	Main Urban Activity
UK1	Sheffield	Students′ Union	53°22′52.09″ N, 1°29′14.57″ E	Tertiary
UK2		West Street	53°22′49.68″ N, 1°28′39.10″ E	Entertainment
UK3		Division Street	53°22′46.65″ N, 1°28′35.98″ E	Entertainment
UK4		Barker′s Pool	53°22′49.42″ N, 1°28′18.58″ E	Commercial
UK5		Leopold Square	53°22′54.21″ N, 1°28′18.52″ E	Entertainment
UK6		Orchard Square	53°22′54.23″ N, 1°28′13.77″ E	Commercial
UK7		Fargate	53°22′52.47″ N, 1°28′10.48″ E	Entertainment
UK8		Peace Gardens	53°22′47.57″ N, 1°28′11.02″ E	Green areas
UK9		The Moor	53°22′32.29″ N, 1°28′26.55″ E	Commercial
UK10		Crookes Valley Park	53°23′1.85″ N, 1°29′37.39″ E	Green areas
UK11		Elmore & Marlborough Road	53°22′52.27″ N, 1°29′51.07″ E	Residential
UK12		Botanical Gardens	53°22′21.44″ N, 1°29′55.31″ E	Green areas
UK13		Fargate cross Black Swan Walk	53°22′56.29″ N, 1°28′6.46″ E	Commercial
UK14		Castle Square	53°22′58.85″ N, 1°27′58.19″ E	Commercial
UK15		Howard Street	53°22′41.21″ N, 1°27′54.38″ E	Tertiary
UK16		St Georges’ Church	53°22′54.61″ N, 1°28′48.26″ E	Tertiary
UK17		Weston Park	53°22′56.17″ N, 1°29′22.82″ E	Green areas
UK18		Headford Gardens	53°22′40.17″ N, 1°28′53.76″ E	Residential
UK19		Bolton Street	53°22′41.50″ N, 1°28′55.72″ E	Residential
UK20		Broomspring Close	53°22′39.47″ N, 1°28′58.42″ E	Residential
UK21		Broomhall Place	53°22′29.85″ N, 1°29′8.33″ E	Residential
UK22		Victoria Road	53°22′28.73″ N, 1°29′12.11″ E	Residential
UK23		Ecclesall Road	53°22′16.06″ N, 1°29′23.37″ E	Commercial
UK24	Doncaster	St Sepulchre Gate	53°31′21.73″ N, 1°8′11.23″ E	Commercial
UK25		High Street	53°31′25.46″ N, 1°8′7.67″ E	Commercial
UK26		Market Place	53°31′28.66″ N, 1°8′2.00″ E	Entertainment
CH1	Tangshan	Community activity area	39°44′6.29″ N, 118°41′31.11″ E	Residential
CH2		Community North Gate	39°44′10.03″ N, 118°41′25.61″ E	Residential
CH3		Shopping centre parking lot	39°44′9.65″ N, 118°41′51.36″ E	Commercial
CH4		South entrance of the square	39°44′12.27″ N, 118°41′50.92″ E	Commercial
CH5		Leisure area near North entrance	39°44′17.32″ N, 118°41′51.14″ E	Entertainment
CH6		North entrance of the square	39°44′19.20″ N, 118°41′50.92″ E	Entertainment
CH7		South entrance of the pedestrian street	39°44′23.94″ N, 118°41′55.59″ E	Commercial
CH8		Middle area of the pedestrian street	39°44′32.12″ N, 118°41′55.30″ E	Commercial
CH9		Middle area of the pedestrian street	39°44′31.83″ N, 118°41′48.95″ E	Commercial
CH10		Middle area of the pedestrian street	39°44′26.27″ N, 118°41′49.67″ E	Commercial
CH11		Market	39°43′53.83″ N, 118°42′24.18″ E	Commercial
CH12		Market	39°43′53.21″ N, 118°42′24.20″ E	Commercial
CH13		Market	39°43′53.86″ N, 118°42′25.41″ E	Commercial
CH14	Beijing	East entrance of the Beijing Old street	39°56′22.49″ N, 116°24′8.96″ E	Commercial
CH15		Bus stop of a street in Beijing	39°56′22.53″ N, 116°24′14.87″ E	Commercial
CH16		Beijing Dongcheng District First Library	39°56′22.85″ N, 116°24′23.41″ E	Tertiary
CH17		Middle island in front of the Orient Plaza	39°54′28.54″ N, 116°24′23.98″ E	Tertiary
CH18		Entrance of Orient Plaza office building	39°54′26.52″ N, 116°24′21.93″ E	Tertiary
CH19		Entrance to Orient Plaza Shopping Mall	39°54′28.61″ N, 116°24′19.88″ E	Commercial
CH20		Wangfujing Avenue	39°54′35.41″ N, 116°24′18.57″ E	Commercial

**Table 2:** Computed indicators for the selected locations of the experiment.

Location ID	*N*	*N*_10_–*N*_90_	*R*	*Fls*	*PEOPLE*	*MUSIC*
UK1	19.85	7.35	2.37	0.0301	80	0
UK2	16.40	10.69	2.13	0.0244	24	0
UK3	15.30	5.47	2.27	0.0153	15	0
UK4	16.75	4.90	2.10	0.0171	60	1
UK5	19.85	4.80	2.45	0.0168	6	0
UK6	15.10	4.03	2.06	0.0125	27	0
UK7	33.75	11.20	2.48	0.0599	117	1
UK8	26.25	3.00	2.62	0.0121	98	0
UK9	23.60	8.05	2.44	0.0456	80	1
UK10	5.96	2.23	1.25	0.0093	4	0
UK11	5.78	3.03	1.16	0.0117	4	0
UK12	6.08	1.10	1.26	0.0066	7	0
UK13	17.80	6.00	2.16	0.0178	117	0
UK14	22.50	4.75	2.51	0.0122	130	0
UK15	16.25	6.35	2.12	0.0209	51	0
UK16	13.60	4.92	2.01	0.0172	28	0
UK17	9.60	1.96	1.64	0.0189	17	0
UK18	7.97	2.01	1.44	0.0067	0	0
UK19	12.65	8.36	2.07	0.0315	34	0
UK20	9.15	4.48	1.56	0.0090	3	0
UK21	10.06	3.75	1.82	0.0105	15	0
UK22	7.87	4.75	1.50	0.0107	15	0
UK23	30.10	18.75	2.93	0.0119	15	0
UK24	18.45	5.15	2.33	0.0195	127	0
UK25	24.50	6.70	2.47	0.0277	126	1
UK26	17.55	7.35	2.22	0.0430	122	0
CH1	6.39	4.33	1.21	0.0211	3	0
CH2	14.25	8.25	2.00	0.0142	14	0
CH3	20.40	8.85	2.13	0.0611	26	0
CH4	19.80	11.60	2.41	0.0571	23	0
CH5	11.85	6.33	1.77	0.0427	42	0
CH6	16.55	18.85	0.79	0.0273	37	0
CH7	23.70	9.85	1.30	0.0329	51	0
CH8	16.80	6.20	0.56	0.0244	57	0
CH9	14.30	4.45	1.87	0.0155	22	0
CH10	16.30	18.88	2.11	0.0159	6	0
CH11	18.70	9.60	2.01	0.0344	54	0
CH12	24.75	11.90	2.45	0.0385	48	0
CH13	16.50	6.65	1.80	0.0342	73	0
CH14	19.60	7.75	2.38	0.0224	34	0
CH15	17.55	16.60	2.27	0.0170	25	0
CH16	17.45	12.00	2.15	0.0174	9	0
CH17	10.19	3.21	1.38	0.0147	11	0
CH18	14.40	6.40	1.92	0.0171	23	0
CH19	18.25	7.30	2.25	0.0270	85	0
CH20	20.70	7.95	2.29	0.0234	142	0

**Table 3:** Main elements contributing to the vibrancy of an urban environment, as coded in the group interview.

Factors (Categories)	Elements (Codes)	Examples of Excerpts from the Group Interview
Aural	Human Voices	*“It sort of implies to me human voices; you can hear some sort of hubbub going on”*
	Variability	*“It is vibrant, it is not stable, it is changing”*
	Loudness	*“It is loud, not quiet”, “You are closer to every sound”, “You feel the vibes…”*
	Music	*“It is like when you have festivals, or funfairs or concerts in the street”*
Visual	People	*“I think vibrancy to me implies people, social context”*
	Activity	*“The railway station is vibrant: many people are walking and going and I think that this helps defining vibrancy with a sort of rhythm”*

**Table 4:** Linear regression model for vibrancy.

Predictor	R^2^ Change	Coefficient (β)
*R*	0.39	0.682
*PEOPLE*	0.15	0.436
*Fls*	0.07	0.383
*N*	0.09	−0.579
*MUSIC*	0.06	0.272
